# Exploring the Link between Plasma Levels of PCSK9, Immune Dysregulation and Atherosclerosis in Patients with Primary Sjögren’s Syndrome

**DOI:** 10.3390/biom13091384

**Published:** 2023-09-12

**Authors:** Vanessa Bianconi, Giacomo Cafaro, Massimo Raffaele Mannarino, Carlo Perricone, Elena Cosentini, Onelia Bistoni, Rita Paltriccia, Rita Lombardini, Roberto Gerli, Matteo Pirro, Elena Bartoloni

**Affiliations:** 1 Unit of Internal Medicine, Department of Medicine and Surgery, University of Perugia, 06123 Perugia, Italy; vanessa.bianconi@unipg.it (V.B.); massimo.mannarino@unipg.it (M.R.M.); elena.cosentini@libero.it (E.C.); rita.paltriccia@unipg.it (R.P.); rita.lombardini@unipg.it (R.L.); matteo.pirro@unipg.it (M.P.); 2 Rheumatology Unit, Department of Medicine and Surgery, University of Perugia, 06123 Perugia, Italy; giacomo.cafaro@unipg.it (G.C.); carlo.perricone@unipg.it (C.P.); onelia.bistoni@ospedale.perugia.it (O.B.); elena.bartolonibocci@unipg.it (E.B.)

**Keywords:** PCSK9, cardiovascular, atherosclerosis, dyslipidemia, inflammation

## Abstract

Proprotein convertase subtilisin/kexin type 9 (PCSK9) regulates lipid metabolism contributing to cardiovascular (CV) risk in the general population. The relationship between PCSK9 and CV risk in systemic autoimmune diseases has been poorly explored. We investigated the association between plasma PCSK9, measures of immune–inflammatory status and markers of atherosclerosis in 52 consecutive patients with primary Sjögren’s syndrome (pSS) in comparison to healthy controls (HCs). Median plasma PCSK9 levels were significantly higher in pSS patients versus HCs (162 (79–255) vs. 53 (39–99) ng/mL). Significantly higher prevalence of subclinical atherosclerosis and lower of dyslipidaemia (61% vs. 85%, *p* = 0.042) characterized pSS patients versus HCs. In pSS, no significant correlation emerged between PCSK9 and disease activity, atherosclerosis and lipid levels. In HCs, PCSK9 significantly correlated with lipid levels and atherosclerosis. Interestingly, significantly higher PCSK9 levels were found in HCs with high-to-very-high as compared to low-to-moderate CV risk (*p* = 0.018) while a non-significant trend towards higher PCSK9 levels was detected in pSS patients with low-to-moderate as compared to high-to-very-high CV risk (*p* = 0.060). This is the first demonstration that pSS patients, despite lower prevalence of dyslipidaemia and higher CV risk profile, are characterized by a 3-fold increase in PCSK9 levels in comparison to HCs. As PCSK9 does not correlate with measures of CV risk, its role in CV morbidity in pSS needs further investigation.

## 1. Introduction

Primary Sjögren’s syndrome (pSS) is a heterogeneous systemic autoimmune disease of unknown etiology characterized by lymphoplasmacellular infiltration of the exocrine glands, responsible for sicca syndrome, and by a wide spectrum of systemic manifestations [1]. Based on several observational studies and meta-analyses, the disease is associated with an increased risk of atherosclerotic cardiovascular (CV) disease [2,3,4,5]. Multiple mechanisms contribute to atherosclerotic damage in pSS. Some traditional CV risk factors, such as hypertension and dyslipidemia, appear to be more prevalent in pSS [6]. Moreover, immune system dysregulation, a hallmark of disease pathogenesis, seems to also be involved. In this regard, circulating anti-Ro/SSA and anti-La/SSB and leukopenia have been associated with different atherosclerosis measures, which suggests their contribution to arterial damage [7,8,9]. Accordingly, pSS patients with leukopenia have been shown to display a 6-fold higher risk of angina [10], while concomitant anti-Ro/SSA and anti-La/SSB positivity has been associated with a higher rate of cerebral infarction in pSS patients [11]. On the other hand, the contribution of systemic inflammation to atherosclerosis in pSS is still uncertain, partly reflecting the autoimmune more than inflammatory pathogenetic background of the disease [12]. Accordingly, several biomarkers of inflammation have been tested as predictors of atherosclerotic damage in pSS patient, with inconclusive results. Among these, calprotectin and Dickkopf-1 (DKK-1) should be further investigated [5,12]. Notably, available evidence suggests that endothelial damage, a crucial player in atherosclerosis development, is an end point of different pro-atherogenic mechanisms and a cumulative index of their joint detrimental action in pSS patients [13]. Accordingly, increased levels of different markers of endothelial cell damage, including endothelial microparticles, soluble thrombomodulin, nitrotyrosine and plasma asymmetric dimethylarginine, have been detected in these patients and have been shown to correlate with disease duration from symptoms and diagnosis [13]. However, the relative contribution of traditional CV risk factors, immune dysregulation and chronic inflammation to the increased risk of atherosclerosis in pSS is still far from understood [14,15,16].

Proprotein convertase subtilisin/kexin type 9 (PCSK9) is a serine protease that plays a crucial role in the regulation of lipoprotein metabolism by promoting the lysosomal degradation of low-density lipoprotein (LDL) receptor (LDLR) on the surface of hepatocytes. With increasing circulating levels of PCSK9 the hepatic LDL clearance is reduced, which promotes atherosclerosis [17,18]. However, compelling evidence suggests that the proatherogenic role of PCSK9 may also be LDL-independent [19,20,21]. In this regard, PCSK9 may act as a regulator of vascular inflammation, leading to the increased recruitment of inflammatory cells into atherosclerotic plaques and a higher risk of plaque destabilization [21,22].

Of note, beyond promoting inflammation, PCSK9 may also be induced by different pro-inflammatory cytokines [23,24]. Accordingly, a pathogenic loop between PCSK9 and inflammation (i.e., systemic inflammation induces PCSK9, which in turn promotes vascular inflammation) may be speculated, which may contribute to accelerated atherosclerosis in patients with rheumatic diseases. In line with this hypothesis, plasma levels of PCSK9 have been demonstrated to directly correlate with disease activity, inflammatory parameters and atherosclerotic damage in patients with chronic autoimmune and inflammatory diseases, including systemic lupus erythematosus (SLE) and rheumatoid arthritis (RA), although with conflicting results [25,26,27,28,29,30,31]. Differences in pathogenic background, mainly autoimmune in SLE and mainly inflammatory in RA, and in the cytokine profile characterizing these two diseases may partly account for such inconsistency. Moreover, concomitant immunosuppressive and corticosteroid therapies may hamper data comparison between studies on SLE and RA [25,26,27,28,29,30,31]. 

To the best of our knowledge, no data are available on PCSK9 in pSS. Interestingly, pSS represents a unique model of systemic autoimmune dysregulation often not requiring immunosuppressive therapies due to its benign course. Thus, it would facilitate the exploration of the crosstalk between PCSK9, autoimmunity and accelerated atherosclerosis. This is an exploratory research aimed at investigating the role of PCSK9 in pSS and its association with disease-specific features and atherosclerotic damage. In particular, the study’s aims were (1) to analyze plasma PCSK9 levels in a cohort of pSS patients free from previous CV in comparison to age- and sex-matched healthy controls (HCs), and (2) to investigate the association between plasma PCSK9 levels and disease-specific parameters of immune dysregulation and measures of atherosclerosis.

## 2. Materials and Methods

### 2.1. Patients and Healthy Controls

In this cross-sectional study, 52 consecutive pSS patients referred to the Rheumatology Unit of the University of Perugia were enrolled. Inclusion criteria included: (1) pSS diagnosis according to American College of Rheumatology (ACR)/European League Against Rheumatism (EULAR) 2016 classification criteria [32], (2) age ≥ 18 years, and (3) written informed consent. Exclusion criteria included: (1) pregnancy/breastfeeding, (2) any previous CV event, (3) estimated glomerular filtration rate (eGFR) < 30 mL/min, (4) cancer diagnosed within the past five years, (5) active acute/chronic infections, and (6) therapy with prednisone equivalents > 10 mg/day in the previous three months.

A group of 26 age- (±5 years) and sex-matched (1:1 ratio) subjects composed of medical and nursing staff members of the Rheumatology and Internal Medicine Units of the “Santa Maria della Misericordia” Hospital of Perugia was included as healthy controls (HCs). 

At inclusion, both pSS patients and HCs underwent physical examination and venous blood sampling after a 13 h overnight fast. Also, for each pSS patient and HC, demographic data, medical history, and concomitant medications were collected from medical records and via interview in the same day as laboratory and instrumental assessment. 

For pSS patients, disease duration was calculated since fulfillment of the ACR/EULAR 2016 classification criteria [32]. Disease activity, disease damage and patient reported outcomes were evaluated at inclusion by the EULAR SS disease activity index (ESSDAI) [33], SS disease damage index (SSDDI) [34] and EULAR SS patient reported index (ESSPRI) [35], respectively. History of extra-glandular manifestations having occurred at any time during disease course was derived from medical records.

### 2.2. Laboratory Parameters

Fasting total cholesterol (TC), HDL-C, triglycerides, creatinine, and glucose were measured using standard procedures. LDL-C was calculated by the Friedewald equation [36]. The “CKD-EPI” equation was used to measure eGFR.

Disease-specific laboratory markers included rheumatoid factor, antinuclear antibodies (ANA), anti-Ro/SSA and anti-La/SSB antibodies, which were measured by nephelometry, immunofluorescent assay, and immunoblotting, respectively.

Plasma PCSK9 was determined by a sandwich enzyme-linked immunosorbent assay method (ELISA; Elabscience Biotechnology Co. Ltd., Wuhan, China); for each plasma sample, two tests were run in two separated plates, and the average of the two observed concentrations was managed as the final concentration (Supplementary Figure S1). High-sensitivity C-reactive protein (hsCRP) was assessed by nephelometry (BN100; Siemens Dade Behring, Siemens S.p.A., Milan, Italy). 

### 2.3. Cardiovascular Variables

Body mass index (BMI) was calculated as weight (kg)/height^2^ (m^2^). Brachial blood pressure (BP) was measured with a sphygmomanometer as previously described [36]. 

The individual ten-year risk of fatal CV disease was estimated according to the 2019 European Society of Cardiology/European Atherosclerosis Society Guidelines for the Management of Dyslipidemias [37]; low-to-moderate CV risk and high–very high CV risk were defined by an estimated ≤4% and ≥5% ten-year risk of fatal CV disease, respectively [37]. Smoking was distinguished between current and previous (≥12 months) habit. Family history of CV disease, defined as the occurrence of a CV event in a male first-degree relative before 55-year age and/or in a female first-degree relative before 65-year age, was recorded. Hypertension was defined by office systolic (S) BP values ≥ 140 mmHg and/or diastolic (D) BP values ≥ 90 mmHg and/or ongoing antihypertensive therapy. Diabetes was defined by at least two fasting blood glucose levels ≥ 126 mg/dL, one HbA1c ≥ 6.5%, and/or ongoing hypoglycemic therapy. Dyslipidemia was defined according to the presence of LDL-C/non-HDL-C levels above the recommended targets according to the individual CV risk, as assessed according to the 2019 European Society of Cardiology/European Atherosclerosis Society Guidelines for the Management of Dyslipidemias [37], and/or ongoing lipid-lowering therapy. Chronic kidney disease was defined by an eGFR ≤ 60 mL/min according to the “CKD-EPI” equation for at least 3 months.

### 2.4. Subclinical Atherosclerosis Parameters

At inclusion, both pSS patients and HCs underwent instrumental evaluation of subclinical atherosclerosis. Brachial artery flow-mediated dilation (bFMD) was assessed by ultrasonography as previously reported [38]. Briefly, after a 10 min rest in the supine position, bFMD measurement was performed for each patient (fast for at least 13 h and not having smoked for at least 8 h) on the non-dominant arm by scanning and measuring with a linear ultrasound probe (frequency between 5 and 10 MHz) the brachial artery diameter before inflation of a pneumatic cuff at 230–250 mmHg for 4 min on the forearm, and after its sudden deflation. The average of three measurements of basal and posthyperemic diameters of the brachial artery was used, and bFMD was calculated as 100 × ((post-hyperemia diameter − basal diameter)/basal diameter) [38]. Aortic pulse wave velocity (aPWV) was assessed non-invasively using an automatic device, the SphygmoCor Vx system (AtCor, Sydney, Australia), as previously described [39]. This technique uses a single-lead ECG and a high-fidelity applanation tonometer to measure the pressure pulse waveform sequentially in two peripheral artery sites, one at the base of the neck for the common carotid artery and the other over the femoral artery. The transit time of the pressure pulse waveform was calculated using the R-wave on the ECG as reference. A graduated caliper was used to calculate the path length (i.e., the distance between the carotid artery site of measurement and the sternal notch subtracted from the distance between the femoral artery site and the sternal notch). Therein, aPWV was calculated using the formula aPWV (m/s) = distance (m)/transit time (s) [39]. Carotid intima-media thickness (cIMT) was assessed by scanning longitudinally the anterior and posterior wall of the common carotid artery, the bifurcation of the common carotid artery, and the internal carotid artery bilaterally with a linear ultrasound probe (frequency between 5 and 10 MHz). For each subject, the ultrasound images were digitally recorded and subsequently analyzed using the AMS II software version 1.1364 [40]. In each carotid segment (1 cm length), both mean (mean) and maximal (max) IMT were evaluated in at least three different frames, and the mean value of the three measurements was chosen. The composite variables cIMT_mean_ and cIMT_max_ refer to the whole (i.e., right + left) carotid tree. cIMT_mean_ is the average of mean cIMT values at six carotid segments (i.e., right common carotid artery, left common carotid artery, right bifurcation of the common carotid artery, left bifurcation of the common carotid artery, right internal carotid artery, and left internal carotid artery), while cIMT_max_ is the greatest value among max cIMT values at the same carotid segments. The presence of carotid atherosclerosis was conventionally defined by the presence of a max cIMT > 1.5 mm in any of the scanned segments.

The study was approved by the local Ethics Committee and was conducted in accordance with current ethical guidelines and regulations.

### 2.5. Statistical Analysis

The SPSS statistical package version 24.0 (SPSS Inc, Chicago, IL, USA) was used for all statistical analyses. The Shapiro test was used to test for normality in the study variables. Categorical variables were expressed as percentages, while continuous variables were expressed as mean ± standard deviation or median (interquartile range). Logarithmic (LG) transformation was performed for skewed variables and the LG-values were used for parametric tests. The independent samples *t*-test, the Mann–Whitney U-test, and the chi-squared test were used for two-group comparisons. Correlation analyses and partial correlation analyses between study variables were performed using the Pearson’s coefficient and the partial correlation coefficient, respectively.

## 3. Results

### 3.1. Characteristics of pSS Patients and HCs

Fifty-two pSS patients (mean age 56 ± 11 years, 92% females) with a median duration of disease of 102 (36–202) months, and 26 age- and sex-matched HCs, were enrolled. All pSS patients had glandular manifestations and 25 (48%) of them extra-glandular manifestations. In total, 43 (84%) and 21 (41%) patients had detectable serum anti-SSA/Ro and anti-SSB/La antibodies, respectively, and 34 (65%) were rheumatoid factor-positive. The median ESSDAI, SSDDI, and ESSPRI scores in the whole cohort of patients were 1 (0–2), 2 (1–2), and 4 (2–7), respectively. In total, 11 (21%) patients were taking hydroxychloroquine, 2 (4%) mycophenolate mofetil, and 2 (4%) low-dose corticosteroid therapy with prednisone < 10 mg/day. One patient was on a treatment with both leflunomide and rituximab. 

**Figure 1:**
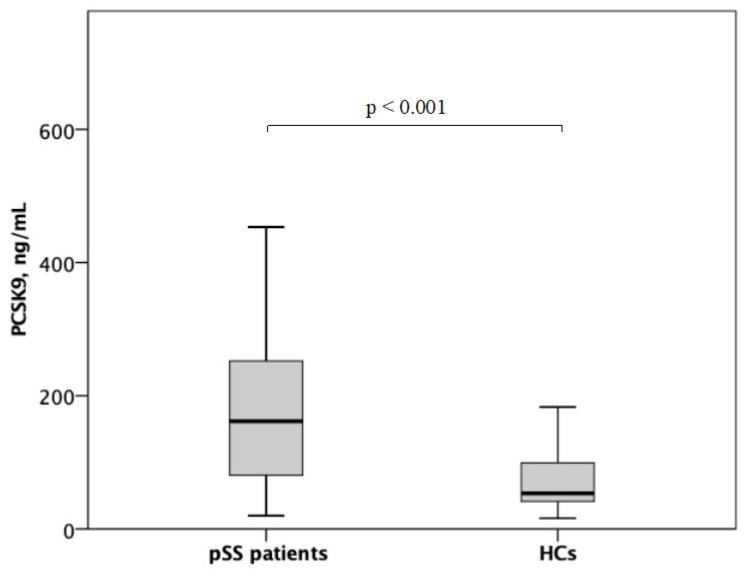
Plasma PCSK9 levels in pSS patients and HCs.

With respect to traditional CV risk factors, a significantly higher prevalence of current smoking habit was detected in pSS patients in comparison to HCs (Table 1). In addition, significantly higher values of SBP and DBP were recorded in pSS patients in comparison to HCs (Table 1). Moreover, pSS patients were characterized by a significantly higher prevalence of family history of CV disease (Table 1). In comparison to HCs, pSS patients were characterized by significantly lower levels of TC, LDL-C, and HDL-C, but displayed significantly higher values of aPWV, as a measure of subclinical atherosclerosis, and an increased prevalence of carotid atherosclerotic plaques (Table 1). Median plasma PCSK9 levels were significantly higher in pSS patients in comparison to HCs (162 (79–255) vs. 53 (39–99) ng/mL) (Figure 1). Characteristics of pSS patients according to the presence of glandular and extra-glandular manifestations are shown in Supplementary Table S1.

### 3.2. PCSK9, Immune-Inflammatory Parameters, and Clinical Features of pSS Patients

**Figure 2:**
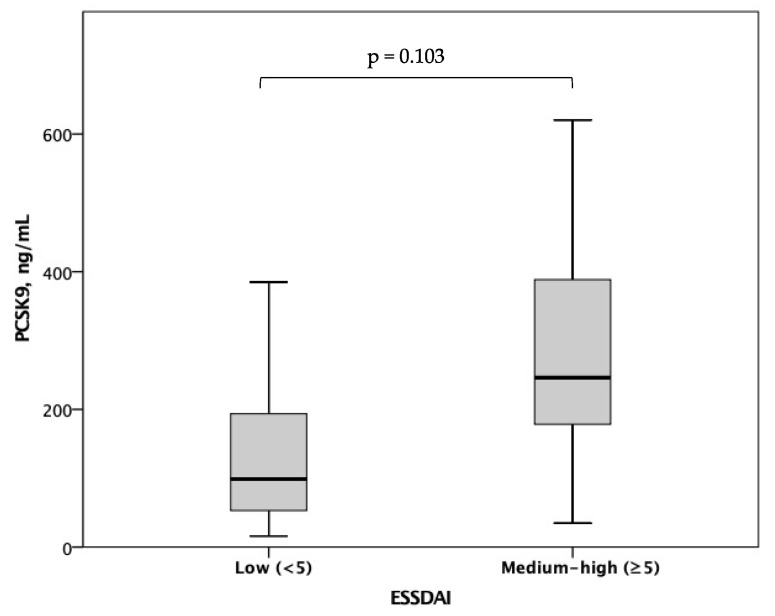
Plasma PCSK9 levels according to low ESSDAI (i.e., <5) versus medium–high ESSDAI (i.e., ≥5) in pSS patients.

In pSS patients, plasma PCSK9 levels did not correlate with hsCRP (r = 0.043, *p* = 0.861), even when accounting for the potential confounding effect of ongoing therapy with either corticosteroids/immunomodulators (r = 0.035, *p* = 0.889) or statins (r = 0.104, *p* = 0.682). No significant correlation was found between plasma PCSK9 levels and either ESSDAI, SSDDI or ESSPRI scores (r = 0.149, *p* = 0.293; r = −0.102, *p* = 0.478; r = 0.014, *p* = 0.923), even when accounting for the potential confounding effect of ongoing therapy with either corticosteroids/immunomodulators (r = 0.147, *p* = 0.305; r = −0.109, *p* = 0.453; r = 0.010, *p* = 0.943) or statins (r = 0.144, *p* = 0.313; r = −0.105, *p* = 0.468; r = 0.014, *p* = 0.922). No significant difference was observed in plasma PCSK9 levels according to each ESSDAI component (i.e., lymphadenopathy, glandular, articular, pulmonary, renal, peripheral nervous system, central nervous system, hematological, biological) (*p* > 0.05 for all comparisons). A trend towards an increase in plasma PCSK9 levels was observed in patients with medium/high (i.e., ESSDAI ≥ 5, the median value in the study population) as compared to those with low disease activity (i.e., ESSDAI < 5) (246 (156–453) vs. 146 (75–238) ng/mL, *p* = 0.103) (Figure 2). There were no significant differences in plasma PCSK9 levels according to circulating anti-SSA/Ro (176 (74–286) versus 134 (74–227) ng/mL, *p* = 0.620), anti-SSB/La antibodies (176 (101–242) versus 144 (76–295) ng/mL, *p* = 0.977), or the presence of extra-glandular manifestations (176 (93–290) versus 146 (72–241) ng/mL, *p* = 0.492). Patients with pSS who were treated with corticosteroids had significantly lower plasma PCSK9 levels as compared to those who were not (29 (26-.) versus 171 (87–265) ng/mL, *p* = 0.006). No significant differences in plasma PCSK9 levels were observed according to ongoing therapy with immunomodulators (201 (107–295) versus 144 (72–245) ng/mL, *p* = 0.164).

### 3.3. PCSK9, CV Risk Factors and Atherosclerosis Burden in pSS Patients and HCs

**Figure 3:**
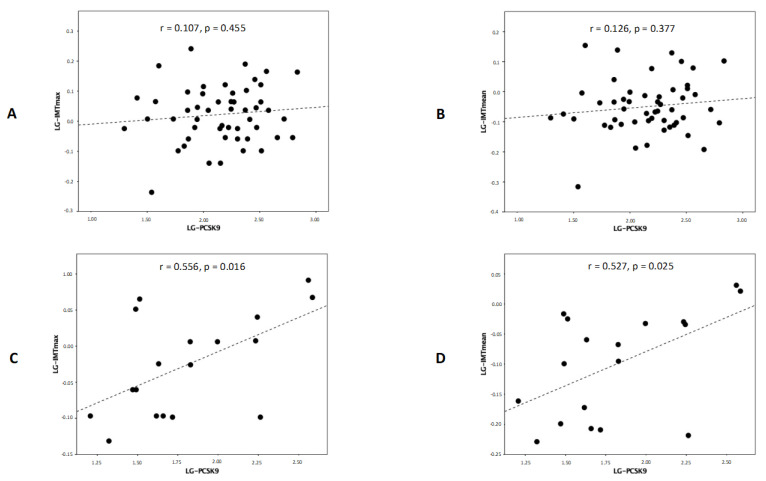
Correlation between PCSK9 and either IMT_max_ or IMT_mean_ in pSS patients (**A**,**B**) and HCs (**C**,**D**).

**Figure 4:**
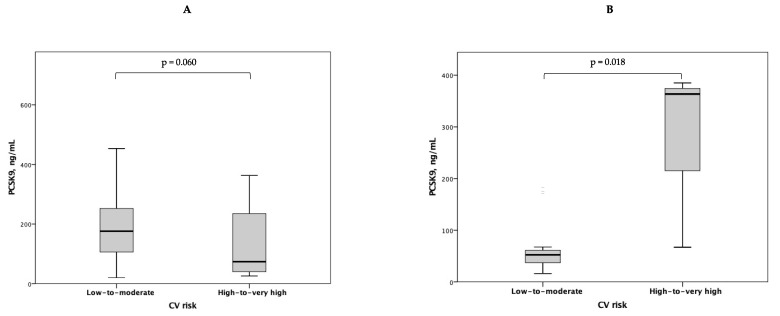
Plasma PCSK9 levels according to low-to-moderate versus high-to-very-high CV risk in pSS patients (**A**) and HCs (**B**).

In pSS patients, no significant correlation emerged between plasma PCSK9 levels and age, BMI, waist circumference, TC, HDL-C, LDL-C, triglycerides, TC-to-HDL-C ratio, SBP, DBP, glucose, or eGFR (*p* > 0.05 for all correlations). On the contrary, in HCs, plasma PCSK9 levels positively correlated with TC (r = 0.457, *p* = 0.019), HDL-C (r = 0.469, *p* = 0.018), LDL-C (r = 0.409, *p* = 0.043), SBP (r = 0.625, *p* = 0.001), and DBP (r = 0.589, *p* = 0.002). There were no differences in plasma PCSK9 levels either in pSS patients or in HCs according to smoking habit, hypertension, diabetes, dyslipidemia, chronic kidney disease, or family history of CV disease (*p* > 0.05 for all comparisons). A significant correlation emerged between plasma PCSK9 levels and either IMT_max_ or IMT_mean_ in HCs, but not in pSS patients (Figure 3). Neither bFMD nor aPWV showed any significant correlation with PCSK9 levels either in pSS patients or in HCs (*p* > 0.05 for all correlations) (Supplementary Figures S2 and S3). In pSS patients, no significant correlation was found between plasma PCSK9 levels and either IMT_max_, IMT_mean_, bFMD, or aPWV, even when accounting for the potential confounding effect of ongoing therapy with either corticosteroids/immunomodulators (r = 0.106, *p* = 0.462; r = 0.128, *p* = 0.375; r = −0.073, *p* = 0.622; r = −0.062, *p* = 0.672) or statins (r = 0.102, *p* = 0.480; r = 0.124, *p* = 0.390; r = 0.054, *p* = 0.714; r = −0.044, *p* = 0.765). Significantly higher plasma PCSK9 levels were found in HCs with high-to-very-high CV risk as compared to those with low-to-moderate CV risk (363 (67-.) versus 52 (33–67) ng/mL, *p* = 0.018), while a non-significant trend towards higher plasma PCSK9 levels was detected in pSS patients with low-to-moderate CV risk as compared to those with high-to-very-high CV risk (176 (100–258) versus 73 (39–264) ng/mL, *p* = 0.060) (Figure 4).

## 4. Discussion

To the best of our knowledge, this is the first study exploring the role of PCSK9, a key regulator of lipid metabolism and a marker of CV risk in the general population, in a cohort of pSS patients free from previous CV events. Our cohort of pSS patients was characterized by a 3-fold increase in plasma levels of PCSK9 in comparison to age- and sex-matched HCs. In pSS patients, plasma PCSK9 levels did not correlate with hsCRP, markers of immune dysregulation or, unlike HCs, with parameters of atherosclerotic damage. Few previous studies have investigated plasma levels and the significance of PCSK9 in systemic rheumatic diseases other than pSS, showing conflicting results [25,26,27,28,29,30,31]. Indeed, while one study demonstrated a significant upregulation of plasma PCSK9 in a cohort of 90 young SLE patients [25], especially in the subgroup with accelerated atherosclerosis and lupus nephritis, other studies have reported either reduced or comparable plasma PCSK9 levels in other SLE cohorts [26,27] or in RA [28], and systemic sclerosis (SSc) patients as compared to HCs [29]. Differences in the clinical features of the enrolled populations may account for such inconsistencies between the aforementioned studies. Nonetheless, whether plasma PCSK9 levels may be somehow influenced by systemic rheumatic diseases remains questionable.

In this regard, the significant direct correlation between PCSK9 and both disease activity and immune–inflammatory parameters, independently of lipid profile, in SLE and RA patients may support a possible relationship between this molecule and the dysregulation of immune–inflammatory pathways [25,26,27,28]. Moreover, in vitro, PCSK9 has been demonstrated to induce the dose-dependent release of pro-inflammatory cytokines by synovial macrophages and synoviocytes in RA patients [30]. However, we failed to demonstrate a significant correlation between plasma PCSK9 levels, hsCRP, autoimmune parameters or disease activity in our pSS cohort. Indeed, pSS represents a chronic disease characterized by a prevalent autoimmune response and by low-grade systemic inflammation. Consistently, in our study, hsCRP levels were comparable between pSS patients and HCs. In line with this point, our finding of lower PCSK9 plasma levels in corticosteroid-treated as compared to corticosteroid-untreated pSS patients is far from being an indirect clue of a possible role of inflammation and immune dysregulation in the upregulation of PCSK9 levels in pSS. Nonetheless, whether corticosteroid therapy may indirectly/directly suppress PCSK9 expression by attenuating immune–inflammatory pathways/downregulating PCSK9 gene transcription needs to be further investigated. Moreover, our pSS patients were characterized by a very low systemic activity, as shown by the median ESSDAI score. Nonetheless, the measurement of disease activity at a single time point does not reflect cumulative activity during disease course. Therefore, any speculation on the relationship between plasma PCSK9 levels and parameters of inflammation, autoimmunity or disease activity in pSS would remain undefined based on our results. 

The relationship between PCSK9 and LDL-C observed in our pSS cohort deserves consideration. In line with previous data, pSSs were characterized by significantly lower LDL-C and HDL-C levels as compared to HCs, despite similar lipid-lowering therapy [41]. This may reflect the cholesterol-lowering effect of systemic inflammation as well as the effect of disease-specific immune-mediated mechanisms. However, unlike the general population [42] and our HCs, PCSK9 levels did not correlate with LDL-C in pSS patients, in whom the upregulation of PCSK9 was observed despite lower circulating LDL-C levels. Thus, it may be hypothesized that PCSK9 upregulation in pSS may reflect a disruption of its physiological role in lipid metabolism in the context of systemic inflammation, and that PCSK9 may exert different biological functions beyond lipid metabolism regulation in patients with autoimmune and inflammatory diseases [24,43,44]. 

In line with data from the literature, our pSS cohort was characterized by an increased atherosclerotic burden as compared to HCs [7,8,45,46,47]. However, in contrast with previous studies [25,28,29], we failed to demonstrate a significant association between PCSK9 levels and any parameter of atherosclerotic damage or CV risk in pSS. Nonetheless, these results are not surprising considering the lack of any correlation between plasma PCSK9 levels and either immune–inflammatory parameters or LDL-C, which are undoubtedly involved as crucial players in the development of atherosclerotic vascular in pSS [45,46]. 

We acknowledge that the present study has intrinsic limitations. First, the cross-sectional design has precluded the possibility of exploring the temporal trend of plasma PCSK9 levels depending on the introduction of corticosteroids/immunomodulators as well as on concomitant modifications in pSS disease activity. Second, the relatively small sample size has reasonably precluded subgroup analyses (e.g., pSS patients receiving versus not receiving corticosteroids/immunomodulators/lipid-lowering drugs or pSS with glandular versus extra-glandular manifestations) and limited the overall reliability of the observed results. In this regard, replication of the study results in larger pSS cohorts, even multicentered ones, would be useful to validate our findings. Third, systemic inflammation was evaluated only through hsCRP. Thus, any correlation between plasma PCSK9 levels and other inflammatory biomarkers in pSS needs to be explored in future studies. 

Overall, until more data are available, no conclusive definition of the role of PCSK9 in pSS can be drawn. However, the study has the strength of providing the first demonstration that pSS patients, despite reduced LDL-C levels, display a significant upregulation of plasma PCSK9 levels in comparison to HCs. 

In conclusion, PCSK9 is upregulated in pSS patients but is not associated with hsCRP levels, disease-specific parameters of immune dysregulation or atherosclerotic burden. Thus, larger longitudinal studies are needed to depict the possible pathophysiological involvement in the disease.

## Figures and Tables

**Table 1:** Characteristics of the study population.

	pSS Patients (n = 52)	HCs (n = 26)	*p*
Age, years	56 ± 11	53 ± 14	NS
Females/Males, n (%)	48 (92)/4 (8)	24 (92)/2 (8)	NS
Family history of CV disease, %	21	0	0.013
BMI, kg/m^2^	25 ± 5	23 ± 4	NS
Waist circumference, cm	91 (85–99)	92 (82–102)	NS
Current smoking, %	21	4	0.046
Previous smoking, %	27	11	NS
Hypertension, %	25	16	NS
Diabetes, %	2	0	NS
Dyslipidemia, %	61	85	0.042
Chronic kidney disease, %	2	0	NS
Carotid plaque, %	10	0	0.038
Low-to-moderate/high-to-very-high CV risk, %	75/25	89/11	NS
IMT_max_, mm	1.09 (0.88–1.23)	1.01 (0.8–1.12)	NS
IMT_mean_, mm	0.87 (0.79–0.99)	0.80 (0.67–0.94)	NS
bFMD, %	6.9 (3.9–12.6)	10.3 (5.1–15.1)	0.054
aPWV, m/s	6.9 (6.1–7.5)	5.5 (5.1–7.1)	0.005
SBP, mmHg	127 ± 14	120 ± 14	0.043
DBP, mmHg	78 ± 7	72 ± 13	0.011
Anti-thrombotic drugs, %	1	0	NS
Lipid-lowering drugs, %	11	8	NS
Statins, %	6	8	NS
Corticosteroids, %	4	0	NS
Immunomodulators, %	27	0	0.004
Anti-SSA/Ro antibodies, %	84	0	<0.001
Anti-SSB/La antibodies, %	41	0	<0.001
Rheumatoid factor, %	66	0	<0.001
ANA, %	100	0	<0.001
TC, mg/dL	195 ± 34	226 ± 32	<0.001
LDL-C, mg/dL	118 ± 30	138 ± 27	0.008
HDL-C, mg/dL	60 ± 12	68 ± 13	0.008
Triglycerides, mg/dL	81 (68–118)	90 (60–116)	NS
TC-to-HDL-C ratio	3.37 ± 0.78	3.41 ± 0.65	NS
Glucose, mg/dL	91 (85–96)	85 (80–95)	NS
eGFR, mL/min	88 ± 22	94 ± 17	NS
hsCRP, mg/L	0.24 (0.00–2.7)	0.12 (0.00–0.72)	NS

Values are expressed as mean ± SD, median (25th–75th percentile), or percentage, as appropriate. Acronyms: ANA, antinuclear antibodies; aPWV, aortic pulse wave velocity; BMI, body mass index; bFMD, brachial artery flow-mediated dilation; HCs, healthy controls; CV, cardiovascular; DBP, diastolic blood pressure; eGFR, estimated glomerular filtration rate; TC, total cholesterol; HDL, high-density lipoprotein; hsCRP, high-sensitivity C-reactive protein; IMT, intima-media thickness; LDL, low-density lipoprotein; PCSK9, proprotein convertase subtilisin/kexin type 9; pSS, primary Sjögren’s syndrome; SBP, systolic blood pressure.

## Data Availability

Data are contained within the article.

## Supplementary Materials

The following supporting information can be downloaded at: https://www.mdpi.com/article/10.3390/biom13091384/s1, Figure S1: ELISA standard curves obtained for measurement of plasma PCSK9 levels in the study population; Figure S2: Correlation between PCSK9 and bFMD in pSS patients (Panel A) and HCs (Panel B); Figure S3: Correlation between PCSK9 and aPWV in pSS patients (Panel A) and HCs (Panel B); Table S1: Characteristics of pSS patients according to the presence of extra-glandular manifestations.
