# Supplementary material for: Exploring the Link between Plasma Levels of PCSK9, Immune Dysregulation and Atherosclerosis in Patients with Primary Sjögren’s Syndrome
Source: Biomolecules. 2023 Sep 12;13(9):1384. doi: 10.3390/biom13091384 (PMC10527459; doi:10.3390/biom13091384)
Supplement: Supplementary file 1 [file biomolecules-13-01384-s001.zip › biomolecules-2484971-supplementary.pdf]

Supplementary Table S1. Characteristics of pSS patients according to the presence of extra-glandular manifestations.

	**Extra-glandular manifestations (n=25)**	**No extra-glandular manifestations (n=27)**	**P**
Age, years	55 ± 10	57 ± 11	0.418
Females/Males, n (%)	23 (92)/2 (8)	26 (96)/1 (4)	0.507
Family history of CV disease, %	32	11	0.065
BMI, kg/m^2	24 ± 4	25 ± 5	0.523
Waist circumference, cm	91 (85-97)	93 (85-102)	0.804
Current smoking, %	16	26	0.381
Previous smoking, %	24	29	0.647
Hypertension, %	20	30	0.423
Diabetes, %	0	4	0.331
Dyslipidemia, %	60	63	0.826
Chronic kidney disease, %	4	0	0.294
Carotid plaque (%)	20	11	0.375
Low-to-moderate /high-to-very-high CV risk, %	72/28	78/22	0.962
IMT_{max}, mm	1.09 (0.94-1.20)	1.01 (0.88-1.25)	0.614
IMT_{mean}, mm	0.87 (0.80-0.97)	0.87 (0.78-1.00)	0.651
bFMD, %	9.5 (5.0-13.5)	5.6 (2.9-7.9)	0.042
aPWV, m/s	6.9 (6.0-7.6)	6.8 (6.1-7.5)	0.977
SBP, mmHg	127 ± 9	127 ± 17	0.958
DBP, mmHg	78 ± 7	78 ± 7	0.901
Anti-thrombotic drugs, %	4	15	0.186
Lipid-lowering drugs, %	4	18	0.102
Statins, %	0	11	0.086
Corticosteroids, %	4	4	0.956
Immunomodulators, %	40	15	0.041
Anti-SSA/Ro antibodies, %	100	70	0.004
Anti-SSB/La antibodies, %	50	33	0.227
Rheumatoid factor, %	75	59	0.234
ANA, %	100	100	1.000
TC, mg/dL	192 ± 37	198 ± 33	0.510
LDL-C, mg/dL	112 ± 28	124 ± 30	0.182
HDL-C, mg/dL	58 ± 12	61 ± 11	0.538
Triglycerides, mg/dL	81 (68-111)	85 (68-126)	0.514
TC to HDL-C ratio	3.34±0.72	3.39±0.85	0.836
Glucose, mg/dL	88 (84-94)	92 (87-105)	0.202
eGFR, mL/min	89 ± 24	87 ± 20	0.675
hsCRP, mg/L	0.21 (0.00-2.64)	0.27 (0.00-2.72)	0.947

Values are expressed as mean ± SD, median (25th-75th percentile), or percentage, as appropriate. Acronyms: ANA, antinuclear antibodies; aPWV, aortic pulse wave velocity; BMI, body mass index; bFMD, brachial artery flow-mediated dilation; HCs, healthy controls; CV, cardiovascular; DBP, diastolic blood pressure; eGFR, estimated glomerular filtration rate; TC, total cholesterol; HDL, high-density lipoprotein; hsCRP, high-sensitivity C-reactive protein; IMT, intima-media thickness; LDL, low-density lipoprotein; PCSK9, proprotein convertase subtilisin/kexin type 9; pSS, primary Sjögren's syndrome; SBP, systolic blood pressure.

A

Standard curve (first plate)	
Optical density	Concentration
2.964	40
1.742	20
1.085	10
0.598	5
0.332	2.5
0.210	1.25
0.128	0.625
0.082	0

B

Standard curve (second plate)	
Optical density	Concentration
3.515	40
2.295	20
1.660	10
1.051	5
0.545	2.5
0.333	1.25
0.197	0.625
0.141	0

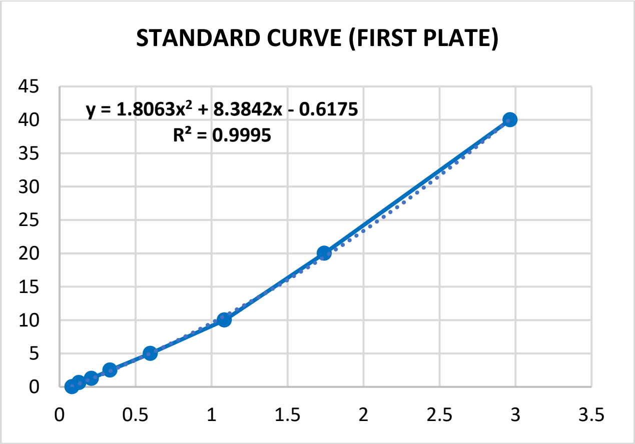

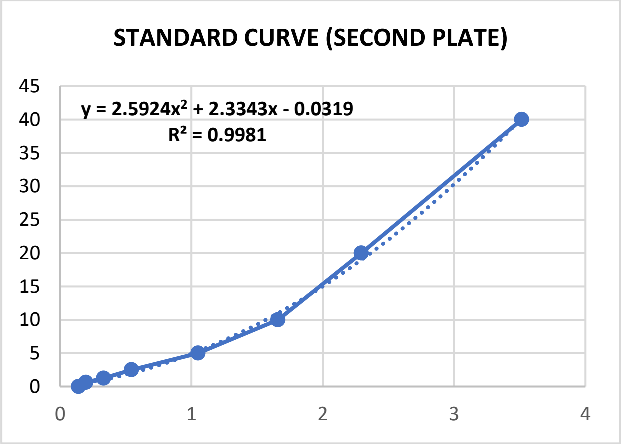

Supplementary Figure S1. ELISA standard curves obtained for measurement of plasma PCSK9 levels in the study population. Panel A: first plate. **Panel B:** second plate.

A

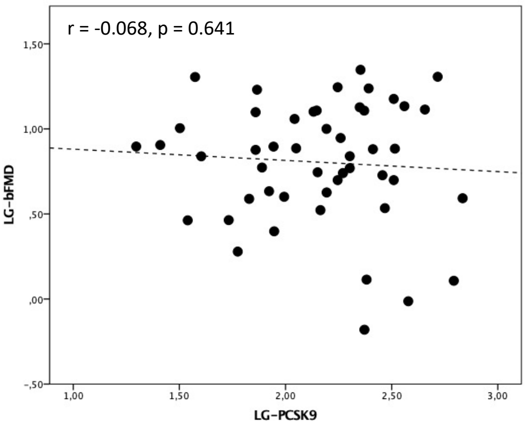

B

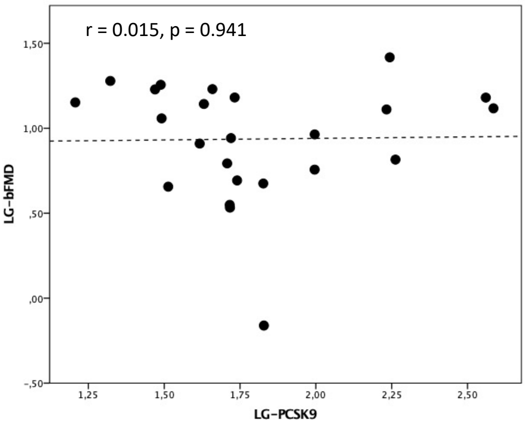

Supplementary Figure S2. Correlation between PCSK9 and bFMD in pSS patients (A) and HCs (B).

A

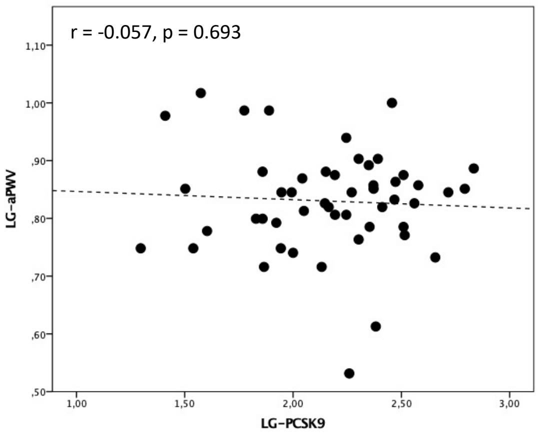

B

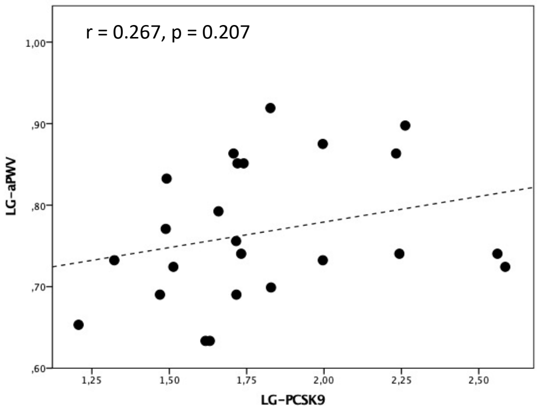

Supplementary Figure S3. Correlation between PCSK9 and aPWV in pSS patients (A) and HCs (B).
